# DNA Repair Capacity and Clinicopathological Characteristics in Puerto Rican Hispanic/Latino Patients with Metastatic Castration-Resistant Prostate Cancer

**DOI:** 10.3390/cancers17020279

**Published:** 2025-01-16

**Authors:** Jaime Matta, Carmen Ortiz-Sánchez, Jarline Encarnación-Medina, Stephanie Torres-Caraballo, Jose Oliveras, Jong Park, Monica M. Arroyo, Gilberto Ruiz-Deya

**Affiliations:** 1 Department of Basic Sciences, Ponce Research Institute, Ponce Health Sciences University, Ponce, PR 00716–2347, Puerto Rico; carmenortiz@psm.edu (C.O.-S.); jencarnacion@psm.edu (J.E.-M.); stetorres@psm.edu (S.T.-C.); joliveras@psm.edu (J.O.); 2 Department of Cancer Epidemiology, H. Lee Moffitt Cancer Center, Tampa, FL 33612, USA; jong.park@moffitt.org; 3 Chemistry Department, Pontifical Catholic University of Puerto Rico, Ponce, PR 00717, Puerto Rico; monica_arroyo@pucpr.edu; 4 St. Luke’s Episcopal Hospital, Ponce, PR 00733, Puerto Rico; gruiz@psm.edu; 5 Department of Surgery, Ponce Health Sciences University, Ponce, PR 00716–2347, Puerto Rico

**Keywords:** Hispanic/Latino (H/L) men, metastatic castration-resistant prostate cancer, indolent, aggressive, clinicopathological variables, Kaplan–Meier curves, DNA repair capacity, nucleotide excision repair, lymphocytes, CometChip assay

## Abstract

Background: Prostate cancer (PCa) accounts for 22% of the new cases diagnosed in Hispanic/Latino (H/L) men in the US. PCa has the highest incidence (38.3%) and mortality (16.4%) among all types of cancer diagnosed in Puerto Rico. We previously showed that PCa patients (*n* = 41) have a significant reduction of 59% in their levels of DNA repair capacity (DRC) when compared to controls (*n* = 14). This study aimed to evaluate DRC levels through the nucleotide excision repair (NER) pathway for the first time in 16 Puerto Rican H/L men with metastatic castration-resistant PCa (mCRPCa) while establishing comparisons with controls and PCa patients with indolent and aggressive disease. Methods: Blood samples and clinicopathological data from PCa cases (*n* = 71) and controls (*n* = 25) were evaluated. PCa cases were stratified into mCRPCa (*n* = 16), aggressive (*n* = 31), and indolent (*n* = 24). DRC levels through NER were measured in lymphocytes with the CometChip assay. The stratification by Gleason score (GS) was GS6 (*n* = 7), GS7 (*n* = 23), GS ≥ 8 (*n* = 20), and mCRPCa patients (*n* = 16). Results: Significant statistical differences were found when comparing the DRC values of the controls with any other of the four PCa patient groups. mCRPCa patients had the lowest mean DRC level of all four patient groups studied. The mean DRC level of mCRPCa patients was 6.65%, and compared to the controls, this represented a statistically significant reduction of 62% (*p* < 0.0001). Further analysis was performed to evaluate the contributions of age, anthropometric measurements, and prostate-specific antigen (PSA) levels to the DRC. Kaplan–Meier curves of mCRPCa revealed that survival probability decreased by approximately 50% by 30 months. This pilot study uses a blood-based phenotypic assay to present the first report of mCRPCa in Puerto Rican men and at a global level of DRC levels of mCRPCa patients. Conclusions: This study evaluated DRC levels through the NER pathway for the first time in 16 Puerto Rican H/L men with mCRPCa. Significant differences in DRC values were found between the controls and the three PCa patient groups. Kaplan–Meier curves revealed that survival probability decreased by approximately 50% by 30 months, and only 20% of the cohort was alive at 50 months, confirming the lethality of mCRPCa in this H/L population. This pilot study represents the first report of metastatic PCa in Puerto Rican men at a global level of DRC levels of mCRPCa patients using a blood-based phenotypic assay.

## Simple Summary

Prostate cancer (PCa) is the most frequently diagnosed cancer type among Hispanic/Latino (H/L) men in the US. Our previous study showed that PCa patients (*n* = 41) experienced a significant reduction of 59% in their DNA repair capacity (DRC) levels when compared to controls (*n* = 14). This study aimed to evaluate DRC levels through the nucleotide excision repair (NER) pathway in a cohort of 16 Puerto Rican (PR) H/L men with metastatic castration-resistant prostate cancer (mCRPCa) using the CometChip assay. A secondary aim was to compare their DRC with three study groups: control, indolent, and aggressive cases. Overall, PCa cases with indolent, aggressive, and mCRPCa had lower DRC values than controls without the disease. Patients with mCRPCa had the lowest DRC levels. The contributions of demographic and clinicopathological factors to DRC were also analyzed. Our data suggest that DRC levels have the potential to discriminate between controls and patients with indolent and aggressive PCa. Kaplan–Meier curves indicated that the probability of survival decreased by approximately 50% within 30 months in mCRPCa patients compared to their initial diagnosis. This pilot study represents a pioneer report of mCRPCa in Puerto Rican H/L men and is the first study to evaluate DRC values of mCRPCa patients using a blood-based phenotypic assay.

## 1. Introduction

Prostate cancer (PCa) is a major health concern, particularly affecting elderly men, and is the second most frequent malignancy in men worldwide and the fourth most common globally [1,2,3,4]. In 2024, approximately 299,010 new PCa cases were diagnosed in the US, according to the American Cancer Society. In 2024, around 35,250 PCa-related deaths occurred in the US. This makes PCa the second leading cause of cancer-related mortality in men in the US. In Puerto Rican men, PCa represents the first cause of mortality of cancer (16%) and incidence (38%) [5]. 

PCa is a complex disease associated with genetic, epigenetic, and environmental risk factors [3,4,6]. Metastatic PCa is classified as advanced-stage PCa [3]. The pathophysiology of metastatic PCa involves the spread of cancer cells through the lymphatic system or bloodstream to distant organs, with common sites of metastasis including bones, lymph nodes, liver, and lungs [3,4,7]. The prognosis for metastatic PCa varies widely, with survival rates heavily dependent on factors such as the extent of metastasis and the patient’s overall health [8,9].

Metastatic castration-resistant prostate cancer (mCRPCa) is a type of metastatic PCa that is defined by a combination of low serum testosterone levels and either biochemical or radiological progression (NCCN Guidelines for Prostate Cancer (2024) (NCCN.org/guidelines, version 4.2024)) [10]. mCRPCa represents a critical and advanced-stage disease characterized by the tumor’s progression despite reduced testosterone (<50 ng/dL) levels achieved through androgen deprivation therapy (ADT) [11]. It is incurable, though various treatments exist and continually evolve to manage symptoms and extend survival [8,12,13,14,15]. The median survival for patients with mCRPCa ranges between 12 and 33 months [16,17,18].

The treatment landscape for mCRPCa is rapidly evolving, with several interventions available [15]. Intensification approaches and treatment sequencing in mCRPCa have been recently reviewed based on 52 clinical trials and ongoing studies [19]. Interventions now include personal treatment history, individual clinical characteristics, symptoms, prognosis, availability of clinical trials, polygenic risk score, prostate-specific membrane antigen-targeted imaging, and newer agents such as poly (ADP-ribose) polymerase (PARP) inhibitors, alone or in combination with androgen receptor pathway inhibitors, radiotherapy, chemotherapy, and targeted therapies such as ^177^Lu-PSMA-617 [20,21]. In recent years, strategies have moved from “one drug and one size fits all” to personalized therapy and intensification with combination therapies [20].

The complexity of mCRPCa is further amplified by its molecular heterogeneity, genomic hallmarks, and the pivotal role of DNA repair mechanisms in its pathogenesis, progression, treatment, and radioresistance. Many PCa studies have highlighted the importance of gene alterations that are responsible for DNA repair [22,23,24,25,26,27]. Dysregulation of at least three DNA repair pathways, including nucleotide excision repair (NER), homologous recombination repair (HRR), and mismatch repair (MMR), has been associated with the complex carcinogenesis process in PCa development and with advanced PCa [23,25,28,29,30,31,32]. Both germline and somatic alterations in DNA repair genes in HRR and MMR are common in approximately 15–25% of mCRPCa patients [6,11,12,29,33].

Defects in DNA repair are critical in driving structural variations in metastatic PCa and represent a potential source for targeted medicine [34,35]. The advent of novel therapeutic agents, such as PARP inhibitors, has transformed the treatment landscape of metastatic PCa, especially for patients with HRR mutations [6,34,36,37]. Despite these advances, there remains a significant lack of understanding concerning the dysregulation of DNA repair pathways in the lymphocytes of PCa patients. Epidemiological studies using functional repair assays in lymphocytes have demonstrated that DRC varies significantly among individuals [38,39,40,41]. Individuals having low DRC levels have a risk factor for the development of several types of cancer [38,42,43,44,45,46,47,48]. Patient stratification based on DNA repair phenotypes may facilitate the identification of which currently FDA-approved drugs, reliant on alterations in DNA repair pathways, may be more beneficial for the patient. This provides an opportunity to explore the use of phenotypic assays that emphasize blood analysis to examine DNA repair in patients with mCRPCa. Evaluating DRC levels in lymphocytes of mCRPCa patients presents a unique research opportunity and contributes to addressing a significant gap in our current understanding of this lethal disease.

Although we have previously reported [30] that the NER pathway is dysregulated in the lymphocytes of patients with indolent and aggressive PCa, it is unknown whether this pathway is also dysregulated in mCRPCa. This pilot study aimed to evaluate the DRC levels in Puerto Rican men with mCRPCa patients for the first time, hypothesizing that mCRPCa patients would have significantly lower DRC levels than controls and patients with indolent and aggressive disease. This represents the first application of a phenotypic assay to study DRC levels in men with mCRPCa.

## 2. Materials and Methods

### 2.1. Study Population and Recruitment

Controls (men without PCa) and pre-operative PCa cases were recruited for this study. The inclusion criteria for controls were men 45 years of age, with normal results from the digital rectal exam (DRE) and normal PSA levels (<4 ng/mL). All PCa cases were pathologically confirmed as primary PCa. Patients were stratified into indolent or aggressive based on the Gleason score (GS): indolent (GS 6 and 7 (3 + 4)) and aggressive (GS 7 (4 + 3) and ≥8). Patients were also classified into five grade groups using their GS [49] according to the NCCN Guidelines for Prostate Cancer (2024) (NCCN.org/guidelines, version 4.2024).

Blood collection was performed at the time of diagnosis before beginning chemotherapy or radiation. For the mCRPCa group, evidence of metastasis was collected to confirm the mCRPCa diagnosis. For this group, medical records were evaluated to collect clinicopathological variables, treatment, and survival status. All participants were recruited either by nurses or physicians in the clinical practice of Gilberto Ruiz-Deya, MD, at San Lucas Hospital in Ponce, Puerto Rico.

### 2.2. Blood Collection

Peripheral blood lymphocytes were isolated from blood samples (6 mL) using BD Vacutainer™ Glass Mononuclear Cell Preparation Tubes (CPTs), Franklin Lakes, NJ, USA. The obtained lymphocytes were stored in 2 mL of freezing media containing 10% dimethyl sulfoxide (DMSO), a 40% RPMI 1640 medium, 50% FBS, and a 1% antibiotic/antimycotic solution. The lymphocytes were stored in a −80 °C freezer for 1–3 weeks. These were thawed in batches of five samples to perform DRC measurements using CometChip (R&D Systems, Minneapolis, MN, USA).

### 2.3. DNA Repair Capacity (DRC) Measurements

These were performed using the CometChip assay (R&D Systems, Minneapolis, MN, USA). The CometChip assay is a high-throughput technology originally developed by Trevigen Inc. (Minneapolis, MN, USA) and published in several studies on DNA damage [50,51,52,53]. This 96-well plate assay allows for measurements of DRC levels with high reproducibility [50]. DRC measurements were performed using the methodology described in detail in a previous study [30]. Briefly, lymphocytes were seeded in 6-well plates and treated with 15 µM aphidicolin C (APC) for 30 minutes before irradiating the cells with 20 J/m^2^ of ultraviolet C (UVC) light. This DNA repair inducer preferentially activates the NER pathway. After allowing two hours of repair, the cells were loaded into the CometChip assay, covered with low melting agarose, and lysed. Alkaline electrophoresis conditions were used to evaluate the level of single-strand DNA damage. After electrophoresis, the chip containing the cells was stained with Yoyo−1^®^, and images were acquired using the EVOS M7000. These images were analyzed using Comet assay software (Trevigen, version 1.3d). A total of 50 comets were evaluated for each experimental condition. The DNA percentage in the tail was used to evaluate single-strand DNA damage. All DRC-level measurements were performed in triplicate for each study participant. The DRC level for each participant was calculated using the equation presented in [54].

DRC = %TD (APC + UVC) − %TD (UVC) − %TD (APC)], where TD is tail density.

### 2.4. Cell Lines

Each DRC measurement experiment included three commercial cell lines as internal controls. Cell lines were purchased from Coriell Institute for Medical Research (Camden, NJ, USA). The GM08925 cell line was included as a model for normal DRC levels. GM02246 and GM02253 cell lines were included as models of medium and low DRC levels since they have knockdowns in XPC and XPD, respectively. DRC values of these three cell lines were published in a previous study [30]. Lymphocytes and cell lines were grown in 88% RPMI−1640, 10% fetal bovine serum (FBS), 1% L-glutamine, 1% antibiotic/antimycotic, and phytohemagglutinin. All cells were grown at 37 °C in a humidified incubator containing 5% CO_2._

### 2.5. Statistical Analysis

Clinicopathological and demographic characteristics (BMI, age of diagnosis, smoking, alcohol use, hypertension, insulin dependency, and key clinicopathological characteristics at diagnosis (PSA, GS, grade group, site, dates of metastasis, medications, vital status, and survival years were grouped by category, frequency, and proportion for all participants using REDCap)) [55]. Cohort distribution by demographic and clinicopathological variables was analyzed using contingency tables and Chi-squared (X^2^) tests. Significance levels were established using a *p*-value cutoff of 0.05 based on a two-tail test for whole comparisons. Analyses were conducted in R using the *ggplot2*, *dplyr*, and Dunn test packages (Ggplot2 version 3.5.1, dplyr version 1.1.4). To compare DRC values across disease types, we stratified the samples into control, non-mCRPCa, and mCRPCa. A Kruskal–Wallis test assessed overall differences in DRC levels among these three disease types. Following a statistically significant Kruskal–Wallis test result (*p* < 0.05), pairwise comparisons were conducted using Dunn’s test with Bonferroni correction to control for multiple comparisons. R packages *ggplot2* and *dplyr* were also used to analyze the distribution of DRC values across GS, specifically within the mCRPCa patient subgroup. DRC values were compared across four categories: control, indolent (GS ≤ 6), aggressive (GS 7–8), and mCRPCa. A final confirmation of mCRPCa diagnosis was obtained by a board-certified urologist (GRD). Box plots were generated to display DRC distribution within each category, with mean values marked on each plot. Groups with significant differences, as identified by Dunn’s test, were marked with an asterisk (*) to highlight statistical significance visually within 95% of CI.

Spearman correlation analysis was performed to assess whether the values obtained at diagnosis of age, BMI, PSA levels, and survival years contributed to the variance observed in DRC values or within clinicopathological variables. Significance levels were established using the *p*-value cutoff of 0.05 based on a two-tail test for the proportions and mean comparisons. All plots, descriptive statistics, one-way ANOVAs, and Tukey’s Honest Significant Different tests were performed using R *reshape2* and *ggplot2* (reshape 2 version 1.4.4) using the methods of Wickham et al. 2016 [56].

### 2.6. Kaplan–Meier Survival Curves

The survival analysis was conducted using the Kaplan–Meier method [57] to estimate the survival probability of patients with the *survival* and *survminer* packages in R. Survival time was calculated as the difference between the date of the last recorded visit and the date of diagnosis, measured initially in months. An analysis was conducted considering survival time in months, and the fitted model provided an estimate of the survival function for the cohort. The Kaplan–Meier survival curve allowed for the depiction of survival probability over time, with the associated confidence intervals.

## 3. Results

### 3.1. Clinicopathological Characteristics of Patients with mCRPCa

The clinicopathological data presented pertain to the time of diagnosis. The mean age of the cohort of 16 patients was 64.6 years (S.E. ± 10.2). The average body mass index (BMI) registered at 39.5 (S.E. ± 4.2), with 63% (*n* = 10) of the patients identified as obese, defined by a BMI of 30 or greater [58]. The mean weight recorded was 206.1 lbs. (S.E. ± 32.2). Furthermore, 44% (*n* = 7) of the patients reported a history of diabetes. The average PSA value at diagnosis was 29.1 ng/dL (S.E. ± 52.1). Notably, PSA values for individual patients exhibited considerable variability over time, influenced by the combined effects of disease progression and treatment interventions (Appendix A).

The median GS was 7, ranging from 6 to 10. Most of the patients were distributed in Grade Group 3. The Gleason grade groups obtained from the core biopsy at diagnosis were grade 1 (*n* = 5), group 2 (*n* = 4), group 3 (*n* = 3), group 4 (*n* = 0), and group 5 (*n* = 4).

Table 1 presents a summary of the data on alcohol consumption, caffeine intake, family history of cancer, diabetes, heart disease, first-degree relative with PCa, radical prostatectomy, and smoking status of the patients in the study.

### 3.2. Treatment

Seven (44%) patients underwent radical prostatectomy. The pharmacological treatment of these patients was complex and evolved throughout the treatment process, primarily due to resistance to specific drugs. Initial treatments included Eligard (Lupron, *n* = 9, 56%), external beam radiation therapy (EBRT, *n* = 4, 25%), abiraterone (Zytiga, *n* = 1, 6%), and finasteride (Proscar, *n* = 1, 6%). One patient (6%) was treated initially with brachytherapy. All four patients (25%) initially treated with EBRT were treated posteriorly pharmacologically with hormone inhibitors (ADT). Twelve patients (75%) were treated with enzalutamide (Xtandi) at some point in their history. One patient (6%) was also treated with chemotherapy docetaxel (Taxotere) and one with apalutamide (Earleada). Considering the first three treatments for all sixteen mCRPCa patients, the most frequent type was hormonal (*n* = 16, 100%), followed by radiotherapy (*n* = 9, 56%). Three patients (19%) did not have a third treatment. The three most frequent therapies used during these initial treatments were Eligard (Lupron, leuprorelin) (*n* = 16, 100%), followed by external beam radiotherapy (EBRT) (*n* = 6, 38%), and Xtandi (enzalutamide) (*n* = 6, 38%). As of the last visit, all patients were on Lupron (leuprorelin) treatment, and seven were on Xtandi.

**Figure 1:**
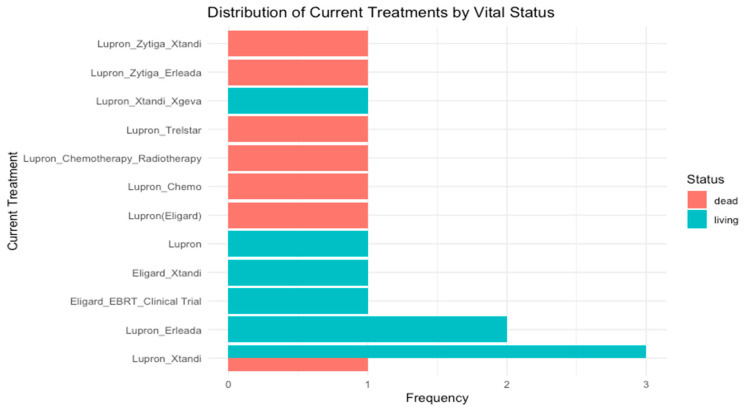
Treatment and vital status of 16 Puerto Rican Hispanic/Latino mCRPCa patients at the time of their last urological evaluation.

Figure 1 summarizes the vital status of the 16 mCRPCa patients according to the distribution of treatments during their last urological evaluation.

### 3.3. DNA Repair Analysis

**Figure 2:**
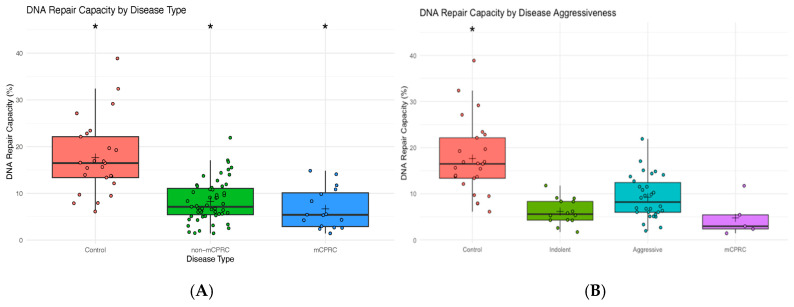
(**A**) Box plots representing the overall DNA repair capacity levels (%) in controls (*n* = 25), non-mCRPCa (*n* = 55), and mCRPCa patients (*n* = 16). (**B**) Overall DNA repair capacity levels (%) in controls and patients with indolent (*n* = 24) or aggressive (*n* = 31) prostate cancer. Symbols represent individual DRC values. The mean DRC value for each group is represented with a plus (+) sign. (*) sign denotes statistically significant differences between groups.

A total of 25 controls and 71 PCa patient samples were analyzed for overall DRC values through NER using CometChip technology. PCa patients were distributed as 16 with mCRPCa, 31 with aggressive disease, and 24 with indolent tumors. The mean DRC value for the control group (*n* = 25) was 17.63%; for all 71 PCa cases, including mCRPCa, it was 6.93% (Figure 2A). This represents a statistically significant reduction in DRC levels of 61% in patients with PCa compared to the controls (*p* < 0.0001, Tukey’s Honestly Significant Difference Test: HSD). The mean DRC value of mCRPCa patients (*n* = 16) was 6.65% (Figure 2A). This represented a statistically significant reduction (*p* < 0.0001, HSD Test) of 62% compared to the controls. The non-mCRPCa patients (*n* = 55) had a mean DRC level of 8.25%, representing a significant reduction of 53% compared to the controls (Figure 2A).

Additional analyses were performed by stratifying PCa patients based on disease aggressiveness using the NCCN guidelines (Figure 2B). Indolent cases included GS ≤ 6 and GS7 (3 + 4), while aggressive cases included GS7 (4 + 3) and GS ≥ 8. Significant differences (*p* < 0.0001, HSD test) were observed when comparing the DRC levels of the control group with the indolent, aggressive, and mCRPCa groups. The mean DRC value for the control group was 17.63%, as previously mentioned. As for the indolent cases, the mean DRC value was 8.51%, while for the aggressive cases, it was 8.43%. Significant differences were found when comparing the DRC levels of the controls with any of the other three groups of PCa patients (Figure 2B). Although mCRPCa patients (*n* = 16) had the lowest (6.65%) mean DRC levels compared to indolent (8.41%) and aggressive cases (8.13%), the difference between the three PCa patient groups was not statistically significant (*p* = 0.56, HSD test) (Figure 2B). No significant differences were observed in disease aggressiveness when comparing the DRC values of patients with aggressive and indolent tumors. However, the case–control difference is still reflected in the results.

**Figure 3:**
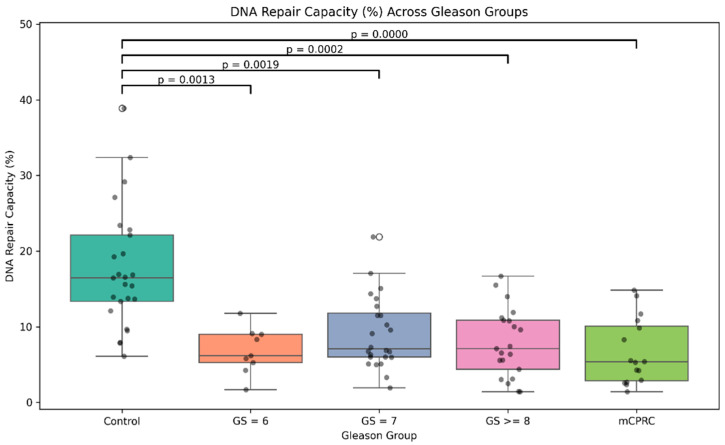
Overall DNA repair capacity in prostate cancer patients and controls. Sample distribution among study groups including controls and prostate cancer cases with tumors with GS 6, GS 7, GS ≤ 8, and mCRPCa. Statistical comparisons between controls and four GS groups and *p*-values are indicated by horizontal lines.

**Figure 4:**
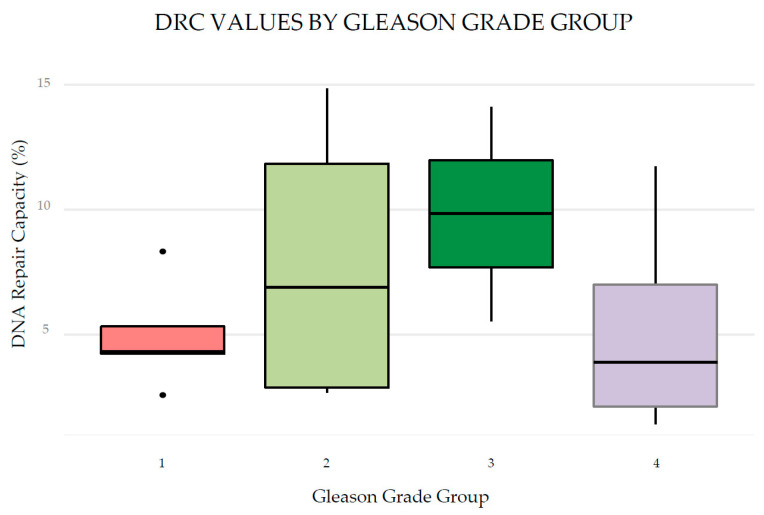
Distribution of DRC values by grade group of 16 patients with mCRPCa according to Gleason score. Grade Groups 1, 2, 3, and 4.

As shown in Figure 3, comparisons were made based on the Gleason score. Significant differences were found when comparing the DRC levels of the controls with any other of the four PCa patient groups (*p* < 0.0001). Specifically, the mean for GS6 was 6.75%, while for GS7, it was 9.14%. For the GS ≥ 8 group, the mean DRC value was 8.08%, and for the mCRPCa group, it was 6.65%. The median DRC values were 16.52%, 6.17%, 7.09%, 7.29%, and 5.53% for controls, GS6, GS7, GS ≥ 8, and mCRPCa patients, respectively (Figure 4).

### 3.4. Distribution of DRC Levels and Correlation with Clinicopathological Variables

Figure 4 shows the distribution of DRC values by Gleason grade group. We did not observe a significant association between DRC and grade groups.

**Figure 5:**
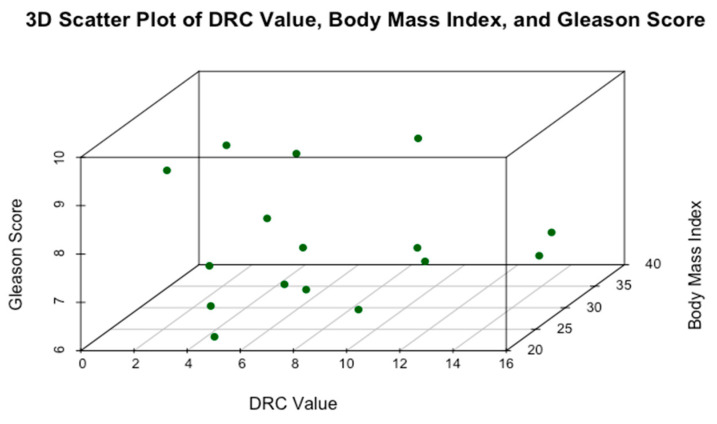
Relationship between DRC, BMI, and GS in 16 mCRPCa Puerto Rican H/L patients.

**Figure 6:**
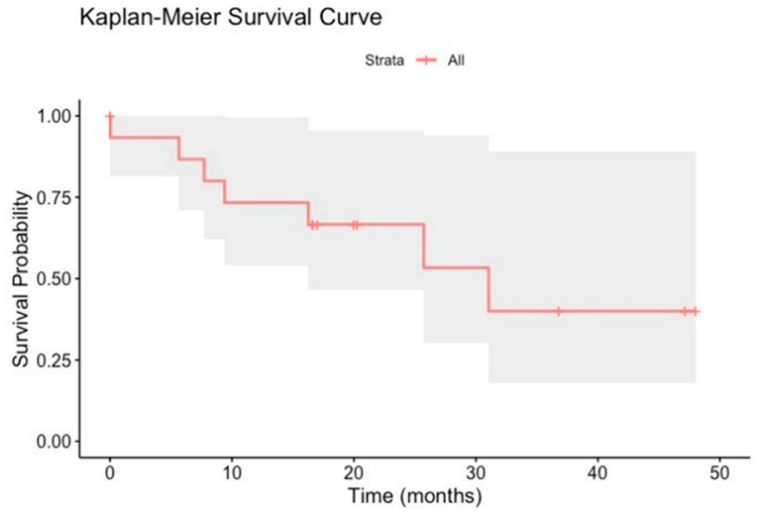
Kaplan–Meier survival curves of 16 mCRPCa patients over 50 months. Flat areas on the curve indicate periods of relatively stable survival. Tick marks on the curve indicate censored patients.

Figure 5 presents a 3D scatter plot of DRC values, BMI, and GS. The 3D scatter plot illustrates the relationship between these variables in 16 mCRPCa patients. DRC values range from approximately 2% to 17% without noticeable clustering in specific regions. No distinct linear trend is apparent between BMI and Gleason scores, suggesting a weak correlation. Patients with high BMIs (<40) exhibit GSs ranging widely from 7 to 10, implying that BMI alone is not a reliable predictor of PCa aggressiveness. Similarly, patients with high GSs (8–10) are distributed across the entire range of DRC values, indicating no clear association between DRC and tumor aggressiveness. Figure 6 also shows instances where individuals with very low DRC values present with either high or low GSs, highlighting that factors beyond DRC and BMI may be associated with tumor development and progression. Overall, the 3D scatter plot suggests that neither DRC nor BMI alone is closely related to GS, underscoring the complexity of PCa aggressiveness and the potential involvement of additional biological factors.

### 3.5. Kaplan–Meier Survival Curves of mCRPCa Patients

The Kaplan–Meier curve of the 16 patients studied visually represents survival over time (Figure 6). The y-axis of the curve represents the survival probability (ranging from 0 to 1), while the x-axis represents time, 50 months.

Figure 6 shows a stepwise decline in survival probability over time, with a notable drop around 500 days (approximately 17 months). This is also the median survival time when survival probability reaches 50%. The shaded region around the curve represents the 95% confidence interval, indicating uncertainty in the survival estimate at each point. Survival probability starts at 1.00 (100%) and decreases to around 0.50 (50%) by approximately 1000 days or 30 months. The Kaplan–Meier curve shows that survival significantly drops around 16.7 months (200 days) and 83.3. months (1000 days) (Figure 6). Around 50 months or 1500 days, the survival probability in the 16 patients with mCRPCa dropped to approximately 20%.

## 4. Discussion

This study represents the first report of advanced-stage, metastatic PCa in Puerto Rican H/L men. At a global level, this pilot study also presents the DRC levels in mCRPCa patients for any ethnic group for the first time. We validated the hypothesis that the men with mCRPCa in our study group had significantly lower DRC levels than the controls. However, no statistical differences regarding DRC levels between mCRPCa patients and those with indolent and aggressive disease were found. The potential differences between these three groups of PCa patients need to be analyzed with a larger sample size and through the homologous recombination repair pathway. This represents the first application of a phenotypic assay to study DRC levels in men with mCRPCa. There is only one previous study in PCa using the CometChip in which we reported a significant reduction in DRC levels in PCa patients with indolent and aggressive tumors [30]. This blood-based approach is in its early stages, and more data from patients of diverse ethnicities are needed before DRC levels can be used as a companion test for treatment decisions.

Epidemiological studies using functional repair assays in lymphocytes have demonstrated that DRC and repair kinetics vary significantly among individuals [39,41,42,45]. Previous studies have also reported that having low DRC levels is a risk factor for the development of several types of cancer [30,38,43,44,45,46,47,48]. However, future studies are needed to identify how DNA repair phenotypes based on lymphocyte DRC levels are associated with mCRPCa progression, including resistance to treatment and the evolution of tumor biology [31].

The CometChip assay has been used to study the repair kinetics of human primary lymphocytes and has identified inter-individual variability in repair kinetics [41]. Identifying individuals with reduced DRC aids in precision medicine [41,50,59]. A meta-analysis performed by Wu et al. 2022 including 55 case–control studies regarding DNA repair phenotypes (including one by this team [38]) and cancer risk found that individuals with the lowest DRC levels had a significantly higher risk of cancer, with pooled odds ratios ranging from 2.02 for skin cancer to 7.60 for liver cancer [47]. The CometChip assay can measure multiple DNA repair pathways [50,59] of multiple cell types and chemical conditions in parallel and integrates them with standard high-throughput screening, high reproducibility, and image analysis technologies [50]. As used in the current study, this assay analyzes the NER pathway’s overall functionality [60]. NER is a versatile pathway responsible for repairing bulky DNA lesions [60,61] and helix-distorting DNA lesions generated by environmental mutagens (including carcinogens) and UV irradiation [62,63]. However, homologous recombination repair (HRR) is the DNA repair mechanism most frequently altered in PCa [29,64]. Poly ADP-ribose polymerase inhibitors (PARPis) have shown antitumor activity and improved overall survival for mCRPCa carrying somatic or germline alterations of HRR [64].

mCRPCa represents a significant challenge in oncology due to its aggressive nature and high risk of progression [12]. Prognostic factors for men with mCRPCa can vary significantly based on the treatment approach, patient health, and other factors. Our study estimated survival using the Kaplan–Meier method [57] with censoring. We identified the exact time when each patient’s death occurred so that each death terminated the previous interval, allowing for a more accurate survival estimate. This analysis showed survival decreased to around 50% after approximately 30 months. Because only 20% of this cohort was expected to be alive at 50 months, early detection of this aggressive phenotype and personalized treatment are essential to prolong survival. Recent studies have reported that Puerto Rican H/L men have worse survival and treatment outcomes than non-Hispanic whites and other Hispanic groups [65,66] Based on Puerto Rican H/L patients (*n* = 642) who were treated after radical prostatectomy at the Veterans Administration Caribbean Healthcare System, this patient population had a significantly higher risk for biochemical recurrence, metastases, development of castration-resistant PCa (CRPC), and PCa-specific mortality (PCSM) [67]. Other studies have also reported that mCRPCa has a notably low 5-year survival rate of approximately 30% [68] since this disease is incurable. More than 90% of mCRPCa patients develop bone metastases, significantly increasing the risk of morbidity [20]. The prognosis for patients with metastatic PCa is influenced by several factors, including the extent of the disease, PSA levels, and the patient’s overall health. The inter-patient variability in multiple factors and comorbidities was evident in this study. This underscores the need for a precision medicine approach in which treatment plans are individually selected for each patient rather than the approach of “one size fits all”.

Patients with rapid PSA doubling times are at greater risk of developing metastatic disease early and dying from their disease [69]. The PSA values of all 16 patients in this study exhibited sharp fluctuations and no discernable pattern. This was perhaps due to the combined effects of advanced-stage disease, multiple treatment modalities, and inter-patient variability. PSA values play a crucial role in managing metastatic PCa by helping to monitor disease progression, assess treatment efficacy, and detect recurrence [69]. However, its limitations necessitate a more holistic approach, including other diagnostic methods and clinical evaluations.

In total, 44% of the mCRPCa in this study had a history of diabetes, and 63% (*n* = 10) of the patients were obese. We are currently investigating the role of metabolic syndrome (MetS) in these patients. Diabetes is one of the five components. MetS syndrome affects 43% of PR H/L adults, with nearly 50% of individuals with elevated glucose [70]. MetS has recently been associated with aggressive PCa but not overall PCa or indolent PCa [58]. Whether MetS is associated with the progression of PCa towards mCRPCa warrants future studies. The association of obesity as a cause of certain diseases, like PCa, can be challenging because of its multifactorial nature derived from genetic, environmental, metabolic, and behavioral factors. These same factors may independently contribute to cancer risk, complicating causal attribution. Some studies suggest a link between obesity and aggressive PCa, while others indicate obesity might be protective against low-grade (indolent) PCa, possibly due to lower testosterone levels [71]. The relationship between obesity and DNA repair is complex, involving multiple biological pathways and factors, including oxidative stress, inflammation, and dietary influences. These factors contribute to the increased risk of DNA damage and cancer in obese individuals. A recent study from a breast cancer case–control cohort of Puerto Rican women showed that, in the controls (*n* = 539), a high BMI was associated with lower DRC levels [72].

This study has limitations that should be considered. First, there was bias in the selection of mCRPCa patients. Patients were selected because mCRPCa represents an aggressive phenotype of PCa that develops due to treatment (ADT). We did not analyze differences in treatment outcomes because the focus was on DRC levels. We were not able to assess the potential confounding effects of treatment in terms of DRC levels. Second, the small sample size may limit the statistical power of some data and warrant future studies with a more significant number of patients, including other non-H/L ethnicities. As reported in other studies [35], difficulty in obtaining samples hinders mCRPCa research and should be accounted for in the study design. Third, although CometChip is a powerful phenotypic tool, the assay cannot identify specific genes in the NER pathway that cause a reduction in DRC levels. Combining phenotypic and genotypic assays can provide a more comprehensive picture of DNA dysregulation and associated risk with PCa. Fourth, the repair pathway measured in this study, NER, termed the “generalist”, could discriminate between controls and PCa cases. It could not distinguish between patients with mCRPCa, indolent, and aggressive disease. However, based on previously published studies [32,73,74], the HRR pathway based on tumor gene expression is associated with high risk [1,6,15,23,27,31]. Our study did not examine DRC levels in lymphocytes regarding HRR pathway activity. This represents an important gap in our current knowledge. Finally, we could not adjust for confounding factors in the Kaplan–Meier curve, making comparing survival differences with other ethnic groups challenging.

## 5. Conclusions

This study evaluated DRC levels through the NER pathway for the first time in 16 Puerto Rican H/L men with mCRPCa. Comparisons were made with controls (*n* = 25) and PCa patients with indolent (*n* = 24) and aggressive (*n* = 31) PCa tumors. Significant differences in DRC values were found between the controls and the three PCa patient groups. Kaplan–Meier curves revealed that survival probability decreased by approximately 50% by 30 months, and only 20% of the cohort was alive at 50 months, confirming the lethality of mCRPCa in this H/L population. This pilot study represents the first report of metastatic PCa in Puerto Rican men at a global level of DRC levels of mCRPCa patients using a blood-based phenotypic assay. This study contributes to ongoing efforts toward developing a blood-based screening test for PCa based on DRC levels.

In the long term, our DRC measurement methodology using the CometChip assay may become a valuable tool for PCa risk prediction based on DRC levels [38] to guide therapy selection to enhance decision-making in precision oncology [73]. Rather than a stand-alone new tool, it can be integrated into genomic, transcriptomic, and epigenomic analyses and clinical information. Future studies are needed to investigate (1) whether DRC levels in mCRPCa patients correlate with treatment responses (e.g., PARP inhibitors or ADT) and their potential as predictive biomarkers, and (2) the application of the CometChip technology to optimize the stratification of mCRPCa patients based on HHR pathway levels, which may benefit most from PARP inhibitors [75].

## Figures and Tables

**Table 1:** Proportion analysis of the study group regarding lifestyle factors, diabetes, family history of cancer, heart disease, and radical prostatectomy.

Factor	No	Yes	Not Reported	Chi^2^	*p*-Value
PCa first-degree relative	13	2	0	7.5943	0.0059
Alcohol consumption	8	6	1	32.0000	<0.0001
Caffeine intake	6	8	1	32.0000	<0.0001
Family history of cancer	9	6	0	32.0000	<0.0001
Diabetes	9	6	0	12.1945	0.0005
Heart disease	3	12	0	10.1091	0.0015
Radical prostatectomy	9	6	0	12.1945	0.0005
Smoking	10	5	0	11.6840	0.0006

## Data Availability

The data presented in this study are available on request from the first author due to the large file.

## Appendix A

The repeated PSA results over time of the 16 mCRPCa in this study are presented in Appendix A.
